# Tezepelumab Improves Small Airways Dysfunction in Severe Asthma: A 52‐Week Real‐World Study

**DOI:** 10.1002/clt2.70147

**Published:** 2026-01-08

**Authors:** Francesco Menzella, Marcello Cottini, Elena Parazzini, Rory Chan, Laura Ventura, Michele Mondoni, Annamaria Bosi, Michela Bortoli, Gianenrico Senna, Carlo Lombardi, Lorenzo Corsi, Silvia Tonin, Andrea Rastelli, Cristina Albrici, Giulia Carone, Maria Sartor, Tatiana Scandiuzzi Piovesan, Maria Rita Marchi

**Affiliations:** ^1^ Pulmonology Unit S. Valentino Hospital Montebelluna (TV) Italy; ^2^ Allergy and Pneumology Outpatient Clinic Bergamo Italy; ^3^ Respiratory Unit, ASST Santi Paolo e Carlo, Department of Health Sciences San Paolo Hospital University of Milan Milan Italy; ^4^ University of Dundee School of Medicine Dundee UK; ^5^ Department of Statistical Sciences University of Padova Padova Italy; ^6^ Department of Pneumology Cittadella Hospital (PD) ULSS6 Euganea Cittadella Italy; ^7^ UOC Allergologia‐ Asma Center University of Verona Verona Italy; ^8^ Departmental Unit of Allergology, Immunology and Pulmonary Diseases Fondazione Poliambulanza Brescia Italy

To the Editor

Small airway dysfunction (SAD) is a critical trait in severe asthma (SA), driving exacerbation risk and oral corticosteroid (OCS) dependence, yet it often remains undetected by conventional spirometry [1]. Advanced techniques, such as oscillometry, now allow for its non‐invasive assessment in clinical practice. Tezepelumab, a monoclonal antibody that blocks the upstream alarmin TSLP, has demonstrated broad efficacy across SA phenotypes by targeting alarmins high up in the inflammatory cascade [2]. However, its long‐term, real‐world impact on SAD remains insufficiently defined except for preliminary remarks [3]. We aimed to evaluate the 52‐week effectiveness of tezepelumab in a cohort of 27 SA patients stratified by SAD and Type 2 (T2) inflammatory status.

This prospective, multicentre, real‐world study included 27 consecutive adult subjects with SA. At baseline, patients were stratified according to the presence of SAD, defined as inspiratory or expiratory reactance values below the lower limit of normal, and by T2 status, with T2‐high defined as blood eosinophil count (BEC) > 150 cells/μL and/or fractional exhaled nitric oxide (FeNO) > 20 ppb. All outcomes were assessed at baseline, 6 and 12 months. Adherence to 4‐weekly subcutaneous administration and follow‐up visits was strictly monitored through hospital pharmacy dispensation records and scheduled clinical visits at baseline, 6, and 12 months.

Statistical analysis were conducted using non‐parametric tests owing to the small sample size (*N* = 27) and non‐normal dat distribution, as assessed by the Shapiro–Wilk test. Differences between independent groups (SAD vs. non‐SAD) were evaluated using the Mann–Whitney *U* test. Associations between categorical variables were analyzed using chi‐squared or Fisher's exact tests. Longitudinal changes across time points (baseline vs. 6 vs. 12 months) were assessed using the Friedman test. Categorical variables were compared using Fisher's exact test. A two‐sided *p* value < 0.05 was considered statistically significant. At baseline, 48% of the cohort exhibited SAD. These patients displayed significantly lower forced expiratory volume in 1 s (FEV_1_) and forced vital capacity (FVC; Supporting Information S1: Table S1), higher total airway resistance (Rrs5), and more abnormal reactance at 5 Hz (Xrs5) and reactance area (AX) compared to the non‐SAD group (Supporting Information S1: Table S2; Table 1).

**TABLE 1:** Sociodemographic and clinical characteristics of patients at baseline and comparison between groups.

Parameter	Overall population	SAD group *N* = 13	Non‐SAD group *N* = 14	*p* value SAD versus non‐SAD	T2‐low *N* = 11	T2‐high *N* = 16	*p* value T2‐high versus T2‐low
Age (years)	60 (39.5; 68)	64 (54; 72)	55 (38; 63)	0.0771	60 (56; 64)	58 (39; 68)	0.6601
Sex (female)	16 (59%)	10 (63%)	7 (50%)	0.7489	10 (77%)	7 (41%)	0.1127
BMI (kg/m^2^)	25.6 (23.2; 29.5)	24.5 (22.6; 29.2)	25.8 (24.1; 28.7)	0.4294	27.8 (24; 30)	25 (23; 28)	0.2168
Smoking status (nonsmoker/smoker)	24 (89%)/3 (11%)	13 (93%)/1 (7%)	12 (75%)/4 (25%)	0.3359	11 (85%)/2 (15%)	14 (82%)/3 (18%)	0.999
Age at asthma onset > 10 years	19 (70%)	9 (69%)	13 (81%)	0.7521	19 (75%)	13 (76%)	0.9999
Switch from other biologics	12 (44%)	7 (44%)	7 (50%)	0.999	6 (46%)	8 (47%)	0.999
Prevalence of SAD (% of patients)	13 (48%)				5 (45%)	8 (50%)	0.9999
Presence of T2 inflammatory status (% high)	16 (59%)	8 (61%)	8 (57%)	0.999			
ACT score	16.0 (12.0; 18.0)	18.0 (15.5; 19.0)	14.0 (12.0; 16.0)	0.0115*	16.0 (13.5; 18.0)	16.0 (8.0; 19.0)	0.8760
AQLQ score	4.2 (4.0; 5.0)	4.2 (3.8; 5.1)	4 (3.3; 4.2)	0.3562	4.0 (3.4; 5.0)	4.1 (4.0; 4.8)	0.6716
BEC (cells/μL)	0.3 (0.1; 45.6)	0.4 (0.1; 90.0)	0.3 (0.1; 23.3)	0.1747	0.1 (0.0; 11.9)	0.4 (0.4; 50.0)	0.0068*
BEC (%)	2.5 (0.9; 4.2)	1.6 (0.5; 4.1)	1.9 (0.9; 2.8)	0.7031	0.5 (0.1; 1.3)	2.3 (1.0; 3.9)	0.0220*
Severe AAER	2.0 (0.5; 3.0)	2.0 (0; 3.0)	2.5 (1.0; 3.0)	0.5667	2.0 (0.0; 4.0)	2.0 (1.0; 3.0)	0.7487
Mild/moderate exacerbations AAER	1.0 (0.0; 2.5)	1.5 (0.7; 2.2)	2.0 (0.0; 2.7)	0.9319	2.0 (0.0; 2.0)	2.0 (0.0; 3.0)	0.7306
OCS (mg/week)	25.0 (18.7; 30.0)	25.0 (5.0; 25.0)	20 (12.5; 32.5)	0.486	25 (18.7; 30.0)	13.7 (5.0; 25.0)	0.1750
IgE (UI/mL)	140.0 (66.5; 756.2)	340.0 (119.0; 925.0)	72.0 (31.0; 206.0)	0.0978	50.0 (24.0; 161.5)	145.0 (117.0; 776.8)	0.0795
FeNO (ppb)	22.0 (12.5; 34.3)	21.0 (13.0; 32.8)	22.7 (10.7; 31.7)	0.5058	12.0 (10.3; 15.0)	32.1 (25.3; 42)	< 0.0001*

*Note:* Data are expressed as absolute frequency (*N* and %) or median (first quartile; third quartile).

Abbreviations: AAER, Annualized Asthma Exacerbation Rate; ACT, Asthma Control Test; AQLQ, Asthma Quality of Life Questionnaire; BEC, Blood Eosinophil Count; BMI, Body Mass Index; FeNO, Fractional Exhaled Nitric Oxide; IgE, immunoglobulin E; OCS, Oral Corticosteroids; SAD, Small Airway Dysfunction.

^*^ Statistical significant.

Treatment with tezepelumab led to an improvement of ACT score by 6 points at 12 months, while the Asthma Quality of Life Questionnaire (AQLQ) score increased by 1.0 unit, both exceeding the minimal clinically important difference by a factor of two (Table 2, Figure S1). Tezepelumab induced a marked reduction in annualized exacerbation rates and maintenance OCS (mOCS) use across all phenotypes (*p* < 0.001). By 12 months, mOCS was discontinued in the majority of patients in both T2‐high and T2‐low groups, a crucial outcome for a cohort historically difficult to treat (Supporting Information S1: Table S3; Figure S2).

**TABLE 2:** Change of clinical characteristics of patients, biomarkers and oscillometry parameters (FOT) over time.

Parameter	Baseline	6 months	12 months	*p* value Friedman
Clinical characteristics and biomarkers				
ACT	16.0 (12.0; 18.0)	23.0 (18.0; 24.0)	22.0 (17.5; 25.0)	0.0001*
AQLQ	4.2 (4.0; 5.0)	5.3 (4.6; 6.0)	5.2 (5.0; 6.0)	0.0001*
BEC (10^9^/L)	0.3 (0.1; 45.6)	0.2 (0.2; 46.7)	0.2 (0.05–19.0)	0.02237*
BEC %	2.5 (0.9; 4.2)	1.3 (0.5; 2.7)	0.2 (0.4; 2.1)	0.1072
FeNO	21.7 (12.2; 33.3)	12.4 (9.2; 24.2)	8.4 (2.2; 13.0)	0.03235*
AAER severe	2.0 (0.5; 3.0)	0.0 (0.0; 0.0)	0.0 (0.0; 0.0)	0.0001*
AAER mild/moderate	1.0 (0.0; 2.5)	0.0 (0.0; 1.0)	0.0 (0.0; 0.0)	0.0019*
OCS (mg/week)	25.0 (18.75; 30.0)	0.0 (0.0; 5.0)	0.0 (0.0; 0.0)	0.0003*
Oscillometry parameters (FOT)				
Rrs5 (cm H_2_O·s/L)	0.4 (0.3; 0.4)	0.3 (0.3; 0.4)	0.4 (0.3; 0.5)	0.184
Rrs5 (% predicted)	124.0 (115.5; 151.5)	120.0 (87.5; 127.5)	98.5 (80.2; 125.0)	0.513
Rrs5‐19	3.1 (2.5; 3.8)	3.1 (2.4; 4.0)	3.2 (2.6; 3.6)	0.744
Xrs5 total *Z*	0.5 (0.0; 1.2)	0.5 (−0.2; 1.0)	−0.7 (−2.6; 0.0)	0.039*
tEFL	0.5 (0.0; 2.0)	0.7 (−0.3; 2.0)	0.6 (0.2; 2.9)	0.308
AX	1.5 (0.1; 3.0)	1.1 (0.5; 2.7)	1.2 (0.5; 2.3)	0.8809

*Note:* Data are expressed as median (first quartile; third quartile).

Abbreviations: AAER, Annualized Asthma Exacerbation Rate; ACT, Asthma Control Test; AQLQ, Asthma Quality of Life Questionnaire; BEC, Blood Eosinophil Count; FeNO, Fractional Exhaled Nitric Oxide; IgE, immunoglobulin E; OCS, Oral Corticosteroids; Rrs5, resistance at 5 Hz; tEFL, tidal expiratory flow limitation; Xrs, reactance; Xrsexp, expiratory reactance; Xrsin, inspiratory reactance; Xrs5, reactance at 5 Hz; Xrs9, reactance at 9 Hz; *Z*, *Z*‐score.

^*^ Statistically significant.

A key finding was the dissociation between spirometric and oscillometric outcomes. While conventional spirometry did not change significantly (Figure S3), reflecting its known insensitivity to peripheral airway changes, oscillometry captured a significant improvement in small airway function (Table 2). This was demonstrated by normalization of Xrs5, with the most pronounced improvements observed in patients with baseline SAD and in the T2‐low subgroup (Supporting Information S1: Table S4). This suggests that tezepelumab effectively targets distal airway pathology, an effect not visible with traditional lung function tests. In parallel, FeNO levels decreased significantly, driven by a strong response in the T2‐high cohort.

Mechanistically, the improvement in SAD may be linked to the broad effects of TSLP blockade. Interleukin (IL)‐13 and IL‐4 are key mediators of small‐airway hyperreactivity [4]. Furthermore, IL‐13 and IL‐5 play an important role in mucus plugging, a key driver of airflow obstruction in SA [5]. In this regard, recent data suggests that mucus plugging is a prominent and heterogenous phenomenon affecting the distal airways in both fatal and non‐fatal asthmatics [6]. By suppressing IL‐4, IL‐5 and IL‐13, it is plausible that tezepelumab improves small airways function through a variety of mechanisms, not only by reducing the downstream production of these specific cytokines and reducing inflammatory damage, but also by improving smooth muscle dysfunction and mucus plugging. Similar improvements have been reported in other recent real‐world studies, which showed results comparable to ours, albeit over shorter observation periods [7, 8].

Our findings suggest SAD as a modifiable “treatable trait” responsive to upstream alarmin blockade. The robust response in T2‐low patients further supports tezepelumab’s role as a broad‐spectrum biologic acting beyond classical T2 pathways. This real‐world study has a number of potential limitations, most notably the relatively small sample size, although it comprised patients who underwent deep clinical phenotyping with oscillometry, which is still seldom performed worldwide. In addition, patients were selected based on clinical need (consecutive adults) rather than randomly, which introduces potential selection bias. However, this limitation is inherent to real‐world study designs. Consequently, a distinction must be made between effectiveness in clinical practice and the causal efficacy established in randomized controlled trials (RCTs), given the absence of randomization and a control arm. Importantly, objective measures such as oscillometry and biomarkers (e.g., FeNO) are less susceptible to placebo effects than subjective questionnaires, thereby strengthening the validity of the present findings.

In summary, these data collected over 12 months complement evidence from RCTs, as causal efficacy can be better inferred from real‐world evidence studies. Furthermore, these findings from more in‐depth clinical phenotyping with oscillometry support the integration of this tool into routine SA management, enabling more informed therapeutic decision‐making and ultimately improving patient outcomes [9].

## Author Contributions

Study conception and design: F.M., M.C. and M.R.M. Collection and interpretation of data: all authors. Manuscript drafting: F.M. Manuscript editing: all authors. Approval to submit: all authors.

## Funding

This project was unconditionally supported by AstraZeneca.

## Ethics Statement

The study was conducted in accordance with the principles of the Declaration of Helsinki and local regulations. This study was approved by the ethics committees of each participating site (Ethics Committee Marca, protocol number 1321/CE, 4 May 2023; Ethics Committee for Clinical Trials of the Province of Padua, protocol number 449, 30 June 2023); and Lombardy Regional Ethics Committee 1 (Protocol No. 2017/ST/246).

## Consent

All patients provided signed informed consent to participate in this study. All patients provided signed informed consent to publication.

## Conflicts of Interest

Dr Menzella reports fees from AstraZeneca, Chiesi, GlaxoSmithKline, Sanofi, and research grants from AstraZeneca, GlaxoSmithKline and Sanofi. Dr Cottini reports personal fees (talks) from Chiesi, Menarini, GSK, and support attending meetings from Chiesi. Dr Chan reports institutional grants from Chiesi, AstraZeneca and GlaxoSmithKline; an advisory board for AstraZeneca; personal fees from AstraZeneca (talks and drafting educational material), personal fees from Chiesi (talks), personal fees from Thorasys (talks) and personal fees from Vitalograph (drafting educational materials); and support attending meetings from AstraZeneca, Chiesi, NIOX, Sanofi‐Regeneron and Vitalograph. Prof Mondoni reports personal fees from Boehringer‐Ingelheim, Chiesi, Sanofi, and AstraZeneca.

## Supporting information


Supporting Information S1

**Figure S1:** Longitudinal assessment of clinical and physiological parameters in the overall severe asthma population.

**Figure S2:** Longitudinal trajectory of clinical outcomes and inflammatory biomarkers stratified by phenotype.

**Figure S3:** Longitudinal evolution of lung mechanics and airflow limitation stratified by phenotype.

## Data Availability

The data presented in this study are available upon reasonable request from the corresponding author.
