# Supplementary material for: Tezepelumab Improves Small Airways Dysfunction in Severe Asthma: A 52‐Week Real‐World Study
Source: Clin Transl Allergy. 2026 Jan 8;16(1):e70147. doi: 10.1002/clt2.70147 (PMC12782232; doi:10.1002/clt2.70147)
Supplement: Supplementary file 1 — Supporting Information S1 [file CLT2-16-e70147-s003.docx]

**Supplementary Table 1: Spirometric characteristics of patients at baseline and comparison between groups.**

| Parameter | Overall population | SAD N=13 | non-SAD N=14 | p-value | T2-low N=11 | T2-high N=16 | p-value |
| --- | --- | --- | --- | --- | --- | --- | --- |
| FEV₁ pre-BD (L) | 1.8 (1.3; 2.7) | 1.2 (0.9; 1.5) | 2.5 (1.8; 3.1) | 0.0018* | 1.5 (1.2; 2.6) | 1.8 (1.6; 2.5) | 0.7304 |
| FEV₁ pre-BD (%) | 69.0 (57.2; 83.5) | 56.0 (43; 63.5) | 80.0 (64.5; 86.5) | 0.0632 | 69.0 (58.0; 83.0) | 63.0 (55.0; 82.0) | 0.7238 |
| FEV₁ post-BD (L) | 1.9 (1.3; 2.6) | 1.7 (1.1; 1.9) | 2.5 (1.9; 3.7) | 0.0169* | 1.7 (1.2; 2.9) | 1.9 (1.4; 2.0) | 0.9999 |
| FEV₁ post-BD (%) | 69.0 (50.0; 78.0) | 58.0 (32.0; 65.0) | 75.0 (67.0; 80.0) | 0.0415* | 64.0 (51.0; 77.5) | 65.0 (45.0; 74.0) | 0.8050 |
| FVC pre-BD (L) | 2.7 (1.8; 3.8) | 1.8 (1.7; 2.2) | 3.3 (2.7; 4.2) | 0.0028* | 2.9 (1.8; 3.3) | 2.7 (2.1; 3.8) | 0.8633 |
| FVC pre-BD (%) | 82.0 (71.0; 92.2) | 67.0 (62.0; 74.0) | 88.0 (77.5; 97.5) | 0.0371* | 85.0 (67.0; 89.0) | 72.0 (65.0; 96.0) | 0.5961 |
| FVC post-BD (L) | 2.9 (2.3; 3.1) | 2.5 (2.2; 2.6) | 3.2 (2.9; 4.8) | 0.0138* | 2.5 (2.2; 4.0) | 2.9 (2.4; 3.0) | 0.9999 |
| FVC post-BD (%) | 77.0 (62.0; 82.2) | 73.0 (59.0; 79.0) | 80.0 (79.0; 100.0) | 0.0485* | 80.0 (73.0; 90.0) | 75.0 (61.0; 81.0) | 0.3968 |
| FEV₁/FVC pre-BD | 67.2 (58.1; 74.5) | 59.2 (55.5; 65.8) | 67.6 (59.6; 75.3) | 0.2463 | 67.6 (58.1; 77) | 63.4 (58.1; 67.6) | 0.9314 |
| FEV₁/FVC post-BD | 65.5 (56.3; 75.7) | 60.4 (55.3; 70) | 66.2 (64.8; 77.8) | 0.1444 | 66.2 (60.7; 72.6) | 64.8 (55.9; 70.8) | 0.7833 |
| tEFL | 0.4 (−0.1; 1.4) | 1.8 (0.7; 3.5) | 0.0 (−0.2; 0.4) | 0.0001* | 1.2 (0.2; 4.2) | 0.4 (−0.0; 1.2) | 0.2318 |
| tEFL % | 0.0 (0.0; 9.6) | 9.2 (0.0; 80.0) | 0.0 (0.0; 0.0) | 0.0014* | 6.7 (0.0; 92.5) | 0.0 (0.0; 6.7) | 0.0703 |

Data are expressed as absolute frequency (N and %) or median (first quartile; third quartile).

**Supplementary Table 2: Oscillometric characteristics (FOT) of patients at baseline and comparison between groups.**

| Parameter | Overall Population | SAD group | Non-SAD group | p-value SAD vs non-SAD | T2-low | T2-high | p-value T2-high vs T2-low |
| --- | --- | --- | --- | --- | --- | --- | --- |
| Rtot pre-BD | 0.4 (0.3; 0.6) | 0.7 (0.7; 1.1) | 0.4 (0.3; 0.4) | 0.0056* | 0.5 (0.4; 0.7) | 0.4 (0.3; 0.7) | 0.9352 |
| Rtot pre-BD % | 151.5 (122.5; 210.5) | 242 (234; 256) | 124 (115; 151) | 0.0050* | 167.0 (124.0; 234.0) | 203.0 (129.0; 234.5) | 0.7551 |
| Rtot post-BD | 0.4 (0.3; 0.7) | 0.5 (0.4; 0.7) | 0.3 (0.3; 0.4) | 0.0416* | 0.4 (0.3; 0.5) | 0.5 (0.3; 0.7) | 0.4380 |
| Rtot post-BD % | 124.0 (107.0; 141.0) | 149.5 (132.5; 191.8) | 119.0 (107.2; 125.5) | 0.2571 | 114.0 (109.5; 127.5) | 126.0 (115.5; 166.5) | 0.5167 |
| Rtot post-BD Chg % | −45.5 (−83.0; −12.0) | −96.5 (−98.0; −83.0) | −15.5 (−33.3; −5.3) | 0.0095* | −53.0 (−77.0; −36.0) | −38.0 (−89.5; −7.5) | 0.5167 |
| Rin pre-BD | 0.4 (0.3; 0.7) | 0.6 (0.5; 1.1) | 0.3 (0.3; 0.4) | 0.0509 | 0.4 (0.3; 0.5) | 0.5 (0.3; 0.7) | 0.4641 |
| Rin pre-BD % | 162.5 (107.2; 227.8) | 216.0 (170.0; 235.0) | 108.0 (107.0; 180.0) | 0.1038 | 136.0 (107.0; 155.0) | 206.0 (139.0; 229.0) | 0.224 |
| Rin post-BD | 0.5 (0.7; 0.3) | 0.5 (0.3; 0.6) | 0.34 (0.3; 0.5) | 0.4707 | 0.3 (0.3; 0.5) | 0.5 (0.3; 0.7) | 0.2982 |
| Rin post-BD % | 117.0 (109.0; 161.0) | 162.5 (135.8; 212.5) | 114.5 (109.0; 150.0) | 0.4762 | 109.0 (103.0; 128.0) | 161.0 (114.5; 205.5) | 0.1833 |
| Rin post-BD chg % | −45.0 (−67.0; −10.0) | −67.5 (−73.0; −59.5) | −10.0 (−36.3; 0.5) | 0.0422* | −46.0 (−67.0; −28.0) | −37.0 (−56.0; −3.0) | 0.4236 |
| Rexp pre-BD | 0.4 (0.4; 0.8) | 1.1 (0.9; 1.2) | 0.4 (0.4; 0.4) | 0.0056* | 0.5 (0.4; 0.9) | 0.5 (0.4; 1.0) | 0.9352 |
| Rexp pre-BD % | 164.0 (139.8; 275.0) | 355.0 (306.0; 396.0) | 148.0 (136.0; 164.0) | 0.0025* | 175.0 (135.0; 295.0) | 215.0 (150.0; 330.0) | 0.6389 |
| Rexp post-BD | 0.4 (0.3; 0.6) | 0.5 (0.3; 0.7) | 0.4 (0.3; 0.5) | 0.4037 | 0.4 (0.3; 0.5) | 0.5 (0.3; 0.6) | 0.8051 |
| Rexp post-BD % | 142.0 (128.0; 174.0) | 183.0 (158.0; 223.0) | 14.00 (130.5; 160.0) | 0.2571 | 128.0 (118.0; 151.0) | 166.0 (140.0; 190.0) | 0.2667 |
| Rexp post Chg % | −27.0 (−48.0; −10.0) | −158.0 (−197.3; −99.7) | −18.0 (−26.7; 0.5) | 0.0191* | −48.0 (−84.5; −37.5) | −26.0 (−115.5; −3.0) | 0.5167 |
| tEFL | 0.4 (−0.1; 1.4) | 1.8 (0.7; 3.5) | 0.0 (−0.2; 0.4) | 0.0001* | 1.2 (0.2; 4.2) | 0.4 (−0.0; 1.2) | 0.2318 |
| tEFL % | 0.0 (0.0; 9.6) | 9.2 (0.0; 80.0) | 0.0 (0.0; 0.0) | 0.0014* | 6.7 (0; 92.5) | 0.0 (0.0; 6.7) | 0.0703 |
| Rrs5 in | 4.0 (2.7; 5.2) | 5.0 (4.5; 5.4) | 2.6 (2.3; 3.6) | 0.0002* | 5.3 (3.3; 5.5) | 4.0 (3.2; 4.6) | 0.0802 |
| Rrs5 Z | 1.0 (-0.5; 2.1) | 2.1 (1.1; 2.8) | −0.6 (−1.3; 0.4) | 0.0008* | 1.1 (0.1; 2.6) | 0.7 (−0.5; 2.1) | 0.3713 |
| Rrs5 % | 133.9 (90.3; 180.7) | 179.8 (134.7; 206.6) | 88.5 (69.8; 111.2) | 0.0008* | 136.3 (104.9; 201.4) | 121.6 (89.4; 179.4) | 0.3965 |
| Rrs5 exp | 4.7 (3.0; 6.2) | 6.2 (5.1; 7.0) | 2.8 (2.6; 4.5) | 0.0005* | 6.3 (4.0; 6.7) | 4.6 (3.4; 5.3) | 0.2233 |
| Rrs5 exp Z | Rrs5 exp Z | 1.6 (0.2; 2.6) | 2.5 (1.7; 3.0) | 0.1 (−0.8; 0.7) | 0.0017* | 1.7 (0.9; 3.2) | 1.6 (0.2; 2.6) |
| Rrs5 exp % | Rrs5 exp % | 152.3 (104.8; 199.5) | 194.5 (156.6; 230.4) | 103.7 (79.5; 121.9) | 0.0010* | 160.1 (129.8; 237.4) | 153.4 (104.2; 201.1) |
| Rrs5 tot | 4.5 (2.8; 5.8) | 5.9 (4.7; 6.4) | 2.7 (2.4; 4.1) | 0.0002* | 5.9 (3.6; 6.3) | 4.4 (3.3; 5.0) | 0.1768 |
| Rrs5 tot (Z) | 1.4 (−0.1; 2.4) | 2.4 (1.7; 2.8) | −0.1 (−1.1; 0.5) | 0.0010* | 1.4 (0.7; 2.8) | 1.5 (−0.1; 2.4) | 0.5991 |
| Rrs5 tot % | 144.5(98.3; 194.8) | 190.9 (156.1; 211.3) | 96.9 (74.5; 117.0) | 0.0010* | 148.2 (122.2; 214.8) | 151.1 (97.6; 195.5) | 0.5991 |
| Xrs5 in | -1.6 (-2.4; -1.1) | −2.4 (−3.2; −2.0) | −1.1 (−1.1; −0.7) | <0.0001* | −1.9 (−2.7; −1.1) | −1.7 (−2.3; −1.1) | 0.5558 |
| Xrs5 Z | −0.9 (−2.9; 0.4) | −2.5 (−4.0; −1.4) | 0.4 (0.2; 0.9) | <0.0001* | −1.0 (−2.4; 0.3) | −0.9 (−3; 0.5) | 0.7666 |
| Xrs5 % | 125.0 (88.2; 208.9) | 195.6 (151.4; 315.8) | 87.8 (72.4; 93.5) | <0.0001* | 126.3 (88.5; 186.4) | 125.7 (84.7; 228.2) | 0.9091 |
| Xrs5 exp | -1.8 (-3.9; -0.9) | −4.3 (−5.8; −3.4) | −0.8 (−1.6; −0.6) | <0.0001* | −3.6 (−6.9; −0.9) | −1.9 (−3.6; −0.9) | 0.2682 |
| Xrs5 exp Z | −2.4 (−5.7; 0.7) | −5.7 (−9.2; −2.8) | 0.7 (−0.4; 1.6) | <0.0001* | −4.1 (−9.7; 0.2) | −2.1 (−4.3; 0.9) | 0.2408 |
| Xrs5 exp % | 169.7 (77.5; 317.2) | 353.7 (221.8; 631.5) | 75.8 (48.9; 112.6) | <0.0001* | 232.6 (93.3; 631.5) | 175.8 (77.9; 282.1) | 0.3965 |
| Xrs5 tot | -1.8 (-3.4; -0.9) | −3.6 (−4.5; −2.9) | −0.9 (−1.3; −0.8) | <0.0001* | −3.1 (−5.5; −0.9) | −1.8 (−3.4; −0.9) | 0.2878 |
| Xrs5 tot Z | -1.8 (-4.3; 0.5) | −4.5 (−6.9; −2.8) | 0.5 (0.03; 1.2) | 0.0001* | −2.9 (−7.4; 0.5) | −1.6 (−3.3; 0.6) | 0.3295 |
| Xrs5 tot % | 137.1 (80.1; 305.9 | 318.1 (200.1; 455) | 80.9 (61.9; 99.6) | 0.0003* | 177.7 (75.7; 450.7) | 158.4 (77.7; 278.1) | 0.6313 |
| AX Z | 1.5 (0.1; 3.0) | 2.5 (1.5; 3.8) | 0.05 (−0.3; 1.5) | 0.0045* | 1.5 (0.1; 3.4) | 2.1 (0.9; 2.7) | 0.7524 |
| AX % | 246.5 (100.4; 691.4) | 639.5 (289.3; 1000.0) | 91.2 (69.0; 158.8) | 0.0003* | 284.5 (103.3; 642.8) | 452.2 (150.6; 697.8) | 0.9999 |
| R5 | 0.9 (0.2; 1.5) | 1.4 (1.1; 1.8) | 0.2 (0.1; 0.4) | 0.0001* | 1.1 (0.2; 1.6) | 0.9 (0.3; 1.2) | 0.4828 |
| Rrs19 in | 2.9 (2.4; 3.6) | 3.4 (2.7; 3.9) | 2.9 (2.1; 2.9) | 0.0318* | 3.7 (3; 4.4) | 2.9 (2.6; 3.0) | 0.0269* |
| Rrs19 Z | -0.0 (−0.4; 0.7) | 0.3 (−0.02; 1.0) | −0.5 (−1.5; 0.03) | 0.0112* | 0.2 (−0.6; 1.6) | 0.0 (−0.5; 0.4) | 0.5676 |
| Rrs19 % | 99.7 (90.3; 120.7) | 109.1 (99.5; 129.9) | 87.6 (68.1; 101.1) | 0.0257* | 105.7 (87; 134.7) | 100.6 (88.9; 110.5) | 0.4780 |
| Rrs19 exp | 3.2 (2.7; 3.9) | 3.6 (3.1; 4.3) | 2.9 (2.3; 3.3) | 0.0372* | 3.9 (2.9; 4.6) | 3.2 (2.9; 3.3) | 0.0924 |
| Rrs19 exp Z | 0.3 (−0.2; 1.3) | 0.9 (0.2; 1.4) | −0.2 (−1.2; 0.3) | 0.0373* | 0.4 (−0.2; 1.7) | 0.3 (−0.2; 1.3) | 0.3965 |
| Rrs19 exp % | 105.7 (94.2; 139.6) | 126.2 (100.2; 144.2) | 94.7 (74.5; 108.8) | 0.0593 | 103.6 (86.9; 154.8) | 107.2 (94.9; 138.9) | 0.6313 |
| Rrs19 tot 5 | 3.1 (2.5; 3.8) | 3.2 (2.7; 3.9) | 3.0 (2.3; 3.8) | 0.1949 | 3.7 (2.5; 4.3) | 3.0 (2.5; 3.3) | 0.0309 |
| Rrs tot 5–19 Z | 0.2 (−0.4; 1.0) | 0.6 (0.1; 1.3) | −0.3 (−1.5; 0.2) | 0.0421* | 0.2 (−0.5; 1.4) | 0.2 (−0.3; 0.9) | 0.5369 |
| Rrs tot 5–19 % | 104.1 (90.7; 131.2) | 117.4 (103.1; 138.3) | 91.7 (69.5; 105.3) | 0.0421* | 104.5 (87.3; 145.3) | 104.2 (92.3; 126.5) | 0.5676 |

Data ara expressed as absolute frequency (N and %) or median (first quartile; third quartile).

**Supplementary Table 3: Comparison of changes of clinical characteristics of patients and biomarkers over time between SAD and non-SAD groups.**

| **Parameter** | **SAD** | | | | **Non-SAD** | | | |
| --- | --- | --- | --- | --- | --- | --- | --- | --- |
|  | **Baseline** | **6 Months** | **12 Months** | **p-value Friedman** | **Baseline** | **6 Months** | **12 Months** | **p-value Friedman** |
| ACT | 18.0 (16.7; 19.0) | 21.0 (20.0; 24.0) | 22.0 (20.0; 25.0) | 0.0072* | 14.0 (12.0; 16.0) | 23.0 (23.0; 24.0) | 24.50 (17.2; 25.0) | 0.0054* |
| AQLQ | 4.8 (4.1; 5.4) | 5.5 (5.0; 6.3) | 5.0 (4.9; 5.7) | 0.023* | 4.0 (3.3; 4.2) | 5.5 (4.7; 6.0) | 6.0 (5.5; 6.1) | 0.0057* |
| BEC (10⁹/L) | 1.1 (0.3; 260.0) | 0.1 (0.2; 60.0) | 0.1 (0.04; 0.2) | 0.2285 | 0.1 (0.1; 0.3) | 3.5 (2.5; 35.2) | 9.2 (0.1; 27.5) | 0.0121* |
| BEC % | 2.9 (0.8; 5.2) | 1.3 (0.7; 3.2) | 1.3 | 0.0821 | 2.1 (0.9; 3.1) | 1.2 (0.5; 2.4) | 2.1 (1.1; 2.2) | 0.5488 |
| FeNO (ppb) | 22.0 (13.0; 35.0) | 20.0 (10.5; 26.0) | 15.0 (9.6; 27.7) | 0.3434 | 22.8 (10.7; 31.8) | 15.4 (9.2; 22.7) | 6.3 (2.3; 9.3) | 0.1354 |
| OCS (mg/week) | 25.0 (5.0; 25.0) | 0.0 (0.0; 0.0) | 0.0 (0.0; 3.7) | 0.0004* | 20.0 (12.5; 32.5) | 0.0 (0.0; 5.0) | 0.0 (0.0; 0.0) | 0.0002* |
| AAER severe | 2.0 (0.3) | 0.0 (0.0; 0.0) | 0.0 (0.0; 0.0) | 0.0014* | 2.5 (1.0; 3.0) | 0.0 (0.0; 0.0) | 0.0 (0.0; 0.0) | 0.0012* |
| AAER mild/moderate | 1.0 (0.0; 2.0) | 0.0 (0.0; 1.0) | 0.0 (0.0; 0.7) | 0.0156* | 2.0 (0.0; 2.7) | 0.0 (0.0; 1.0) | 0.0 (0.0; 0.0) | 0.0019* |
|  | **T2-low** | | | | **T2-high** | | | |
| ACT | 16.0 (12.5; 17.5) | 23.0 (18.5; 24.7) | 24.0 (22.5; 24.7) | 0.0038* | 16.5 (11.0; 19.0) | 23.0 (20.5; 24.0) | 23.5 (15.7; 25.0) | 0.0050* |
| AQLQ | 4.2 (3.0; 5.0) | 5.3 (4.7, 6.0) | 6 (5.0; 6.0) | 0.0094* | 4.1 (4.0; 4.9) | 5.6 (5.0; 6.1) | 5.2 (4.7; 6.1) | 0.0027* |
| BEC (10⁹/L) | 0.1 (0.0; 0.5) | 0.2 (0.0; 30.0) | 0.03 (0.01; 0.04) | 0.1561 | 0.3 (0.1; 277.5) | 0.2 (0.2; 50.0) | 0.3 (0.1; 27.5) | 0.4493 |
| BEC % | 0.7 (0.5; 1.8) | 0.4 (0.1; 1.1) | 0.3 (0.1; 0.4) | 0.2167 | 3 (2.5; 4.4) | 2 (1.0; 4) | 1.5 (0.6 – 2.1) | 0.1072 |
| FeNO (ppb) | 12.0 (9.6; 14.0) | 11.0 (6.7; 20.7) | 6.8 (2.6; 16.0) | 0.8777 | 32.8 (25.1; 48.5) | 19.1 (10.9; 28.0) | 9.3 (7.2; 29.5) | 0.0354* |
| OCS (mg/week) | 25.0 (18.7; 30.0) | 0.0 (0.0; 2.5) | 0.0 (0.0; 0.0) | 0.0008* | 13.7 (5.0; 25.0) | 0.0 (0.0; 0.0) | 0.0 (0.0; 0.0) | 0.0001* |
| AAER severe | 2.0 (0.5; 3.5) | 0.0 (0.0; 0.0) | 0.0 (0.0; 0.0) | 0.0012* | 2.0 (0.7; 3.0) | 0.0 (0.0; 0.0) | 0.0 (0.0; 0.0) | 0.0002* |
| AAER mild/moderate | 1.0 (0.0; 2.0) | 0.0 (0.0; 0.5) | 0.0 (0.0; 0.0) | 0.0742 | 1.5 (0.0; 3.0) | 0.0 (0.0; 0.7) | 0.0 (0.0; 0.0) | 0.0004* |

**Supplementary Table 4: Comparison of oscillometry (FOT) parameters over time between SAD and non-SAD and T2-low and T2-high groups.**

| **Parameter** | **Baseline** | **6 Months** | **12 Months** | **p-value** | **Baseline** | **6 Months** | **12 Months** | **p-value** |
| --- | --- | --- | --- | --- | --- | --- | --- | --- |
|  | **SAD** | | | | **Non-SAD** | | | |
| Rtot pre-BD (kPa/L/s) | 0.7 (0.7; 0.9) | 0.7 (0.5; 0.7) | 0.5 (0.5; 0.8) | 0.0969 | 0.4 (0.3; 0.4) | 0.3 (0.3; 0.4) | 0.4 (0.3; 0.5) | 0.1835 |
| Rin pre-BD | 0.7 (0.6; 0.9) | 0.6 (0.5; 0.7) | 0.6 (0.5; 0.8) | 0.0969 | 0.3 (0.3; 0.4) | 0.3 (0.3; 0.3) | 0.3 (0.3; 0.4) | 0.999 |
| Rexp pre-BD | 0.9 (0.9; 1.0) | 0.7 (0.5; 0.7) | 0.6 (0.6; 1.0) | 0.2636 | 0.4 (0.4; 0.4) | 0.3 (0.3; 0.4) | 0.4 (0.3, 0.5) | 0.3114 |
| Xrs5 in | -2.5 (–3.3; –2.3) | -2.5 (–2.8; –2.1) | -2.5 (–2.9; –1.71) | 0.0930 | -1.1 (-1.1; -0.7) | -1.0 (-1.3; -1.0) | -1.4 (-1.9; -1.0) | 0.2008 |
| Xrs5 exp | -4.0 (–6.1; –3.1) | -3.5 (–4.5; –2.7) | -3.3 (–4.8; –1.9) | 0.0439* | -0.8 (-1.6; -0.6) | -0.8 (-1.2; -0.6) | -1.8 (-4.0; -1.2) | 0.0330* |
| Xrs5 Total | -3.5 (–5.0; –2.7) | -2.9 (–3.8; –2.6) | -3.2 (–3.9; –1.8) | 0.0439* | -0.9 (-1.3; -0.8) | -0.9 (-1.2; -0.8) | -1.7 (-3.1; 1.2) | 0.0335* |
| Xrs Total Z | -4.3 (–8.4; –2.8) | -3.9 (–4.7; -2.1) | -2.7 (–4.9; –1.3) | 0.0439* | 0.5 (0.0; 1.2) | 0.5 (-0.2, 1.0) | -0.7 (-2.4; -0.0) | 0.0385* |
| AX in Z | 2.6 (1.4; 3.8) | 2.7 (1.1; 3.6) | 2.6 (1.6; 3.5) | 0.8825 | 0.0 (-0.3; 1.5) | 0.5 (-0.2; 0.9) | 0.7 (0.4; 1.8) | 0.6144 |
| Rrs5–19 Total | 3.3 (3.0; 5.1) | 3.5 (2.9; 4.0) | 2.6 (2.5; 3.1) | 0.4169 | 2.9 (2.2; 3.2) | 2.6 (2.0; 4.0) | 3.3 (3.2; 4.1) | 0.8357 |
|  | **T2–low** | | | | **T2–high** | | | |
| Rtot pre-BD (kPa/L/s) | 0.4 (0.3; 0.5) | 0.4 (0.3; 0.4) | 0.5 (0.4; 0.6) | 0.5488 | 0.4 (0.3; 0.6) | 0.3 (0.3; 0.4) | 0.5 (0.3; 0.5) | 0.0407* |
| Rin pre-BD | 0.4 (0.3; 0.5) | 0.4 (0.3; 0.4) | 0.4 (0.3; 0.5) | 0.7788 | 0.4 (0.3; 0.7) | 0.3 (0.3; 0.3) | 0.45 (0.3, 0.5) | 0.1653 |
| Rexp pre-BD | 0.5 (0.4–0.6) | 0.4 (0.3; 0.5) | 0.5 (0.4; 0.7) | 0.7788 | 0.4 (0.4; 0.8) | 0.3 (0.3; 0.4) | 0.55 (0.4; 0.6) | 0.0743 |
| Xrs5 in | -1.8 (–3.1; –1.1) | -1.5 (–2.5; –1.0) | -1.3 (–2.1; –1.1) | 0.2359 | -1.6 (-2.3; -1.0) | -1.8 (-2.2; -1.1) | -1.9 (-2.2; -1.5) | 0.8111 |
| Xrs5 exp | -2.3 (–9.5; –0.9) | -1.4 (–4.3; –0.8) | -1.8 (–3.4; –1.3) | 0.0051* | -1.8 (-3.6; -0.8) | -2.3 (-3.0; -0.4) | -3.0 (-4.6; -1.8) | 0.4037 |
| Xrs5 Total | -2.0 (–7.8; –1.0) | -1.4 (–3.6; –0.8) | -1.6 (–2.9; –1.2) | 0.0091* | -1.8 (-3.1; -0.8) | -2.1 (-2.7; –1.1) | -2.8 (-3.6; -1.7) | 0.4037 |
| Xrs Total Z | -1.3 (–8.6; –0.5) | -0.0 (–3.3; -0.7) | -0.6 (–2.0; –0.2) | 0.0211 | -2.0 (-3.6; 0.3) | -1.4 (-4.1; -0.5) | -2.6 (-5.0; -1.1) | 0.4037 |
| AX in Z | 1.3 (-0.1; 2.1) | 0.7 (0.0; 1.5) | 0.57 (0.4; 1.2) | 0.8669 | 2.18 (1.0; 3.6) | 1.2 (0.6; 3.2) | 1.4 (0.9; 3.3) | 0.9770 |
| Rrs5–19 Total | 3.8 (2.6; 4.2) | 3.7 (2.3; 4.2) | 3.3 (3.1; 4.1) | 0.3679 | 3.0 (2.6; 3.2) | 2.9 (2.5; 3.8) | 2.8 (2.5; 3.3) | 0.2105 |

Data are expressed as absolute frequency (N and %) or median (first quartile; third quartile). *Statistically significant.

AAER: Annualized Asthma Exacerbation Rate; ACT: Asthma Control Test; AQLQ: Asthma Quality of Life Questionnaire; AX: area of reactance; BEC: Blood Eosinophil Count; FeNO: Fractional Exhaled Nitric Oxide; FEV₁: Forced Expiratory Volume in one second; FEV₁/FVC: Ratio of Forced Expiratory Volume in one second to Forced Vital Capacity; FVC: Forced Vital Capacity; IgE: immunoglobulin E; OCS: Oral Corticosteroids; Post-BD: post-bronchodilator; Pre-BD: pre-bronchodilator; Rexp: expiratory resistance; Rin: inspiratory resistance; Rrs: resistance; Rrs5: resistance at 5 Hz; Rrs9: resistance at 9 Hz; SA: severe asthma; SAD: small airway dysfunction; T2: type 2; tEFL: tidal expiratory flow limitation; Tot: total; Xrs: reactance; Xrs5: reactance at 5 Hz; Xrs9: reactance at 9 Hz; Xrsin: inspiratory reactance; Xrsexp: expiratory reactance; Z: Z-score.

**Figure legends**

**Supplementary figure 1: Longitudinal assessment of clinical and physiological parameters in the overall severe asthma population.**

This figure displays the aggregate changes across three time points (Baseline, 6 months, and 12 months) for asthma control (ACT) and quality of life (AQLQ), inflammatory biomarkers (BEC, FeNO), and lung mechanics measured via spirometry and Forced Oscillation Technique (FOT).

**Supplementary figure 2: Longitudinal trajectory of clinical outcomes and inflammatory biomarkers stratified by phenotype.** This figure illustrates the evolution of clinical status and type 2 inflammation markers over 12 months (Baseline, 6 months, 12 months). Panels compare:

*Clinical Control*: Asthma Control Test (ACT) scores, Asthma Quality of Life Questionnaire (AQLQ), Oral Corticosteroid (OCS) consumption, and Annualized Asthma Exacerbation Rates (AAER).

*Biomarkers*: Blood Eosinophil Count (BEC) and Fractional Exhaled Nitric Oxide (FeNO). Comparisons are stratified by the presence of Small Airway Dysfunction (SAD vs. Non-SAD) and inflammatory profile (T2-high vs. T2-low).

**Supplementary figure 3: Longitudinal evolution of lung mechanics and airflow limitation stratified by phenotype.** This figure displays the changes in lung function parameters from Baseline to 12 months, comparing SAD vs. Non-SAD groups and T2-high vs. T2-low groups.

*Oscillometry (FOT):* Evolution of Total Resistance (Rtot), Inspiratory/Expiratory Resistance (Rin, Rexp), Reactance at 5 Hz (Xrs5), Total Reactance (Xrs Total), and Area of Reactance (AX).

*Spirometry:* Changes in Forced Expiratory Volume (FEV₁) and Forced Vital Capacity (FVC). Data highlights statistically significant differences in the response to treatment between phenotypes regarding small airway resistance and reactance.
