# Supplementary figures and images for: Tezepelumab Improves Small Airways Dysfunction in Severe Asthma: A 52‐Week Real‐World Study
Source: Clin Transl Allergy. 2026 Jan 8;16(1):e70147. doi: 10.1002/clt2.70147 (PMC12782232; doi:10.1002/clt2.70147)

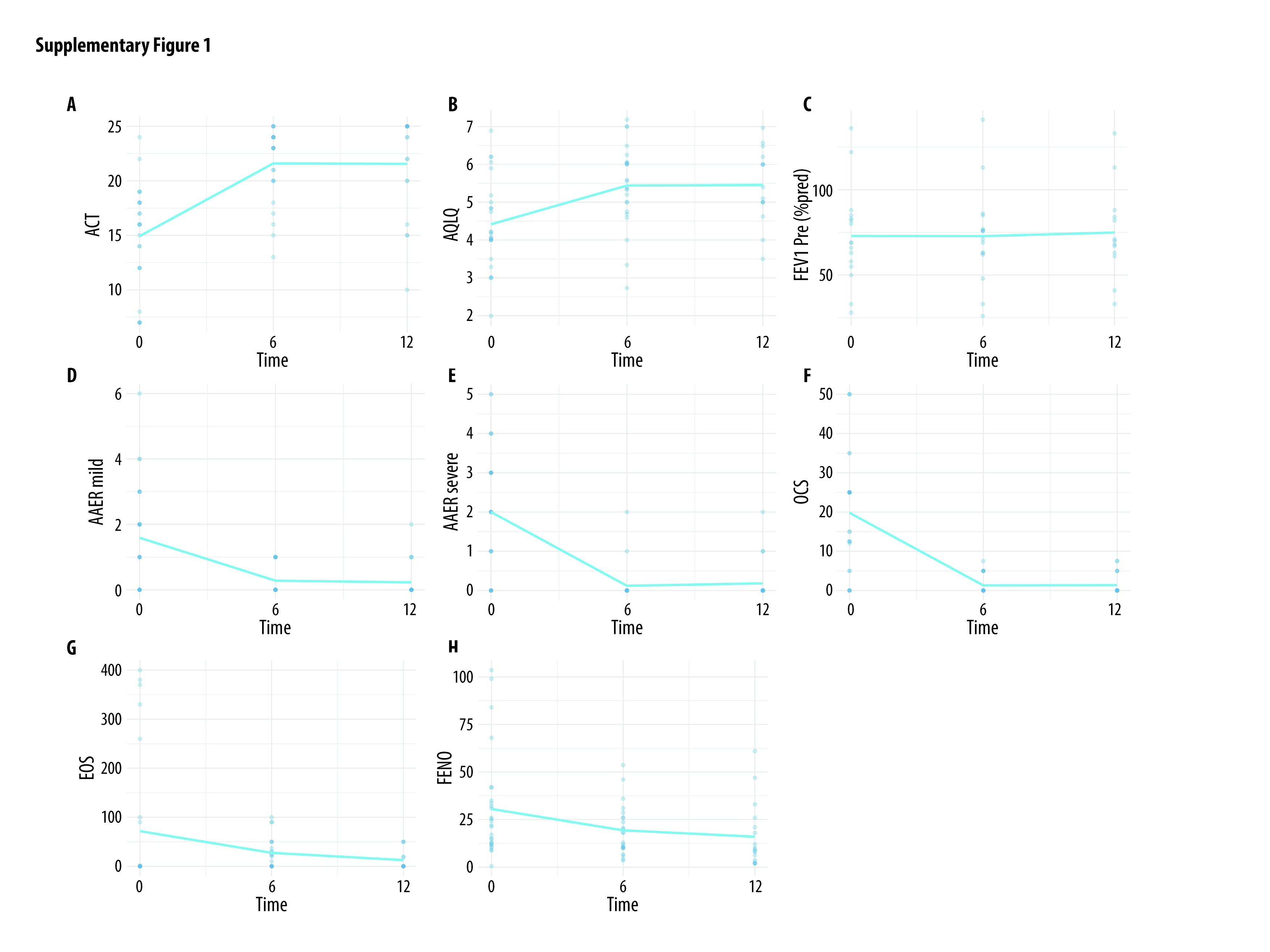

Supplement: Supplementary file 2 — Figure S1: Longitudinal assessment of clinical and physiological parameters in the overall severe asthma population. [file CLT2-16-e70147-s002.tif]

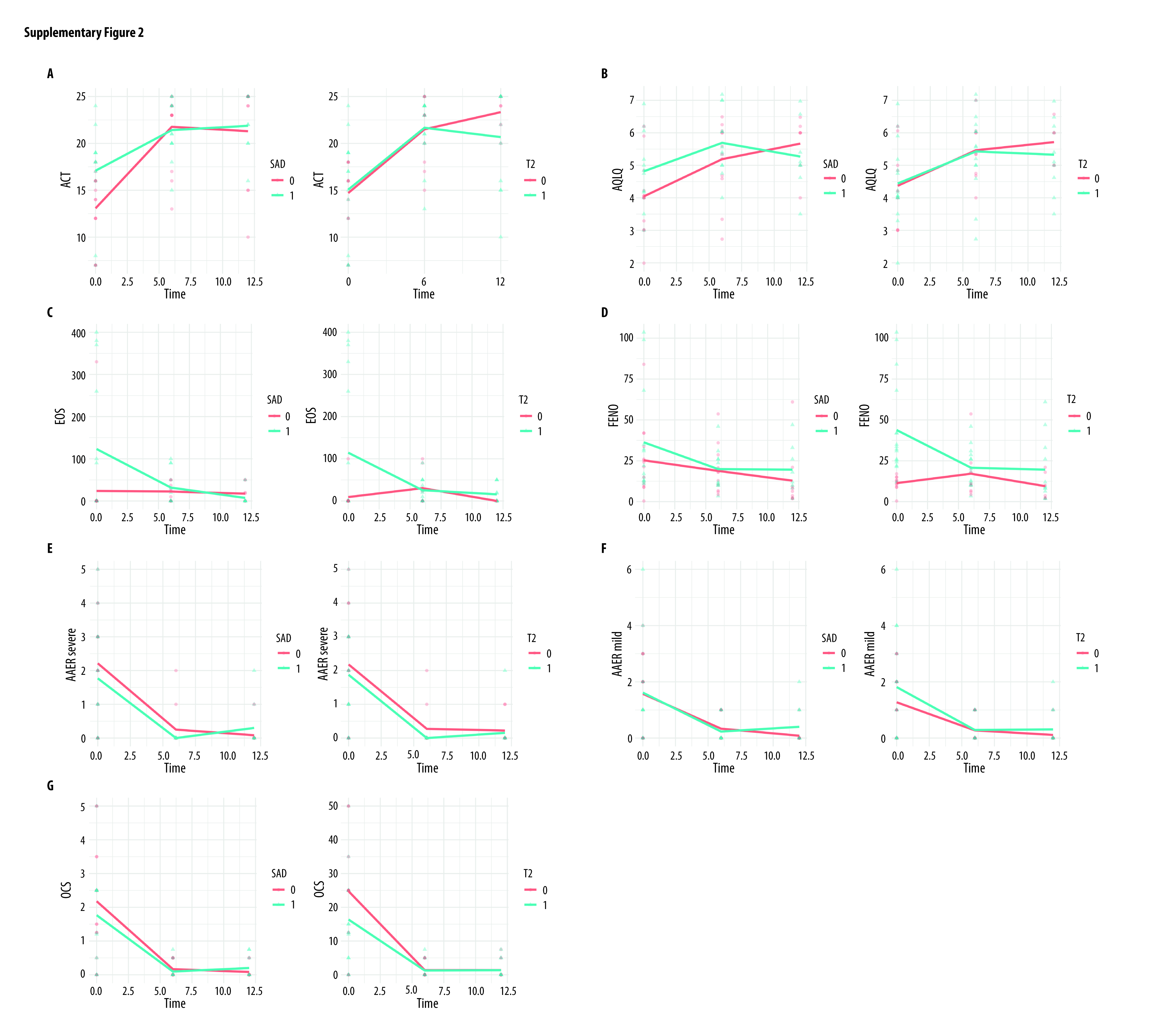

Supplement: Supplementary file 3 — Figure S2: Longitudinal trajectory of clinical outcomes and inflammatory biomarkers stratified by phenotype. [file CLT2-16-e70147-s001.tif]

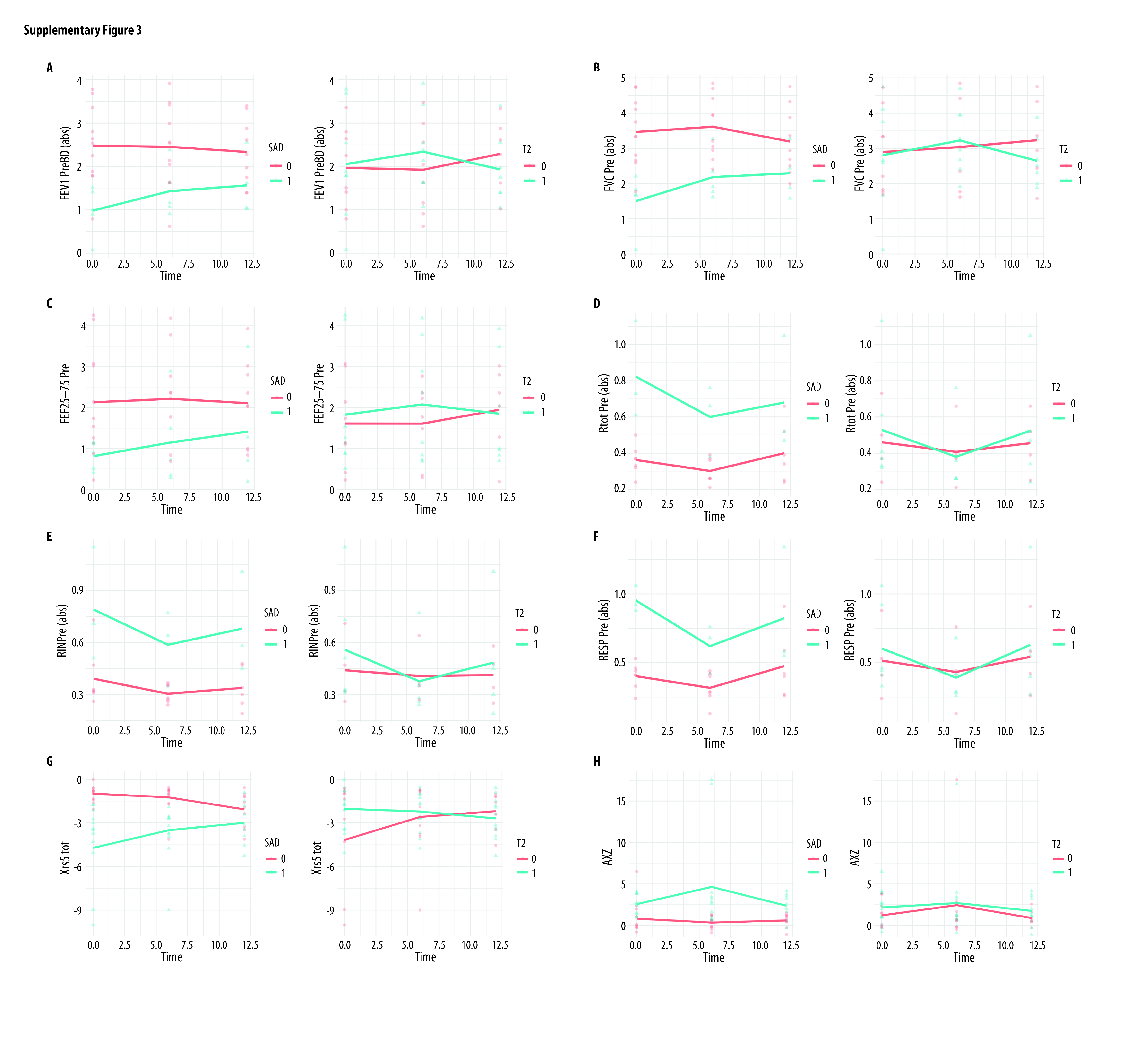

Supplement: Supplementary file 4 — Figure S3: Longitudinal evolution of lung mechanics and airflow limitation stratified by phenotype. [file CLT2-16-e70147-s004.tif]
